# High-dose thiotepa-related neurotoxicity and the role of tramadol in children

**DOI:** 10.1186/s12885-018-4090-6

**Published:** 2018-02-13

**Authors:** Christophe Maritaz, Francois Lemare, Agnes Laplanche, Sylvie Demirdjian, Dominique Valteau-Couanet, Christelle Dufour

**Affiliations:** 1 Department of Clinical Pharmacy, Gustave-Roussy cancer campus, 114 Rue Edouard Vaillant, 94805 Villejuif, France; 2 Department of Biostatistics and Epidemiology, Gustave-Roussy, Villejuif, France; 3 Department of Pediatric and Adolescent Oncology, Gustave-Roussy, Villejuif, France; 4 Faculty of Pharmacy of Paris, Sorbonne-Paris University, 75 006 Paris, France; 5 EA 7348 MOS, Ecole des Hautes Etudes en Santé Publique, 35000 Rennes, France

**Keywords:** Thiotepa, Neurotoxicity, Tramadol, Pediatrics

## Abstract

**Background:**

Serious neurological adverse events (NAE) have occurred during treatment with high-dose thiotepa regimens of children with high-risk solid tumours. The objective was to assess the incidence of NAE related to high-dose thiotepa and to identify potential contributing factors that could exacerbate the occurrence of this neurotoxicity.

**Methods:**

From May 1987 to March 2011, children with solid tumours treated with high-dose thiotepa were retrospectively identified. Each NAE detected led to an independent case analysis. Potential contributing factors were pre-specified and univariate/multivariable analyses were performed.

**Results:**

Three hundred seven courses of thiotepa (251 patients) were identified. The total dose per treatment ranged from 600 to 900 mg/m^2^. 81 NAE (26%) were identified. 46 NAE were related to high-dose thiotepa during the first course (18.3%) and 11 during the second course (19.6%). The symptoms appeared in a median time of 2 days after the introduction of thiotepa. Central and peripheral symptoms were headaches, tremors, confusion, seizures, cerebellar syndrome, and coma. High-dose thiotepa was reintroduced in 18 cases and symptoms reappeared in 5 children. For 3 patients who had seizures during the first course, premedication with clonazepam for the second course has prevented recurrence of NAE. As contributing factors, brain tumour and tramadol treatment increased the risk of thiotepa-related neurotoxicity by 2 to 6 times respectively.

**Conclusions:**

The incidence of neurotoxicity was 18.3%. Brain tumours and tramadol treatment are risk factors to consider when using high-dose thiotepa. The outcome of patients was favourable without sequelae in all cases and rechallenge with thiotepa was possible.

## What is already known about this subject

- Little is known about the possible relationship between exposure to high-dose thiotepa and the occurrence of neurological disorders.
- High-dose thiotepa administered below the Maximum Tolerated Dose has never been studied in terms of neurological outcomes.

## What this study adds

- This study estimates the incidence of thiotepa-related neurological complications, unlike the neurotoxicity described when the drug was used beyond the defined limiting dose or combined with another cytotoxic drug or irradiation.
- Our findings show that brain tumour and tramadol treatment could be independently associated with thiotepa-related neurotoxicity.

## Background

High-dose chemotherapy (HDCT) with autologous stem cell transplantation (ASCT) has improved the survival of children with high-risk solid tumours. The rationale for HDCT is that escalated doses of HDCT may increase survival by capturing putative remnant malignant cells [1]. The rationale for ASCT following HDCT is a planned rescue for HDCT-related severe haematological toxicity [2].

The HDCT regimens are generally based on the use of alkylating agents [3]. Among them, thiotepa (N,N′,N″-triethylenethiophosphoramide), an organophosphorus compound with the formula SP(NC_2_H_4_)_3_, is one of the few drugs to have demonstrated clear activity at high doses in childhood tumours [4–9]. Thiotepa is extensively metabolized by cytochrome P450. Its major metabolite tepa, has similar alkylating activity [10, 11]. This hepatic biotransformation is mediated by CYP3A4 and CYP2B6, and conjugation is catalyzed by glutathione S-transferase (GST) [12, 13]. Following intravenous administration, drug exposure in cerebrospinal fluid (CSF) is almost equivalent to that of the product in plasma [14]. Thiotepa has been reported to possess an elimination half-life of 1.3–5.2 h and tepa an elimination half-life of 3–21 h [15]. Thiotepa and its metabolites are totally excreted in urine [15, 16]. Due to its metabolic profile, concomitant administration of thiotepa with CYP3A4 or CYP2B6 inhibitors may increase plasma thiotepa concentrations and potentially decrease the concentrations of the active metabolite tepa. Conversely, concomitant administration of thiotepa with CYP3A4 or CYP2B6 inducers may reduce plasma thiotepa concentrations and increase those of tepa. In addition, there is interindividual variability in product excretion since CYP3A4, CYP2B6, and GST share a genetic polymorphism with some variants which exert a significant impact on the clearance of thiotepa and tepa [17]. Relationships between the pharmacokinetics of the drug and its toxicity have been described [18–21]. Toxicity to the gastrointestinal and central nervous system is dose-limiting and the maximum tolerated dose (MTD) of thiotepa is between 1005 and 1125 mg/m^2^ [7, 21, 22].

Little is known about the possible relationship between exposure to high-dose thiotepa and the occurrence of neurological disorders. To our knowledge, high-dose thiotepa administered below the MTD has never been studied in terms of neurological outcomes. One reason for the lack of knowledge is that thiotepa is rarely used alone. In our experience, serious neurological adverse events (NAE) have occurred during treatment with high-dose thiotepa regimens. Some of these NAE could lead to life-threatening complications. The objective of the present study was to assess the incidence of NAE related to high-dose thiotepa with ASCT in children with solid tumours and to identify potential contributing factors that could increase the occurrence of this neurotoxicity.

## Methods

### Patients

The institutional review board of the Gustave Roussy Cancer Campus approved this study. The parents/guardians gave their written informed consent for the retrospective analysis of clinical data according to the institutional review board of the Gustave Roussy Cancer Campus. From the institutional pediatric transplantation register, we established a retrospective cohort of all patients treated for a solid tumour with high-dose thiotepa followed by ASCT between May 1987 and March 2011 at Gustave-Roussy, center with expertise in the treatment of complex malignancies. Our analysis focused on neurotoxicity exclusively due to thiotepa, therefore patients treated with thiotepa combined with another cytotoxic drug or radiotherapy were excluded.

### Procedures

All medical and nursing records were reviewed. Demographics data (sex, age, weight, height), race aimed at assessing the influence of the type of metabolism (slow, medium, fast), clinical data (medical and neurological history, allergies, cancer type and site of the primary tumour), and drug exposure (thiotepa and drugs administered concomitantly) from admission to hospital until the day of ASCT were collected for each patient. To assess the impact of potential organic disturbances, biological monitoring of renal and hepatic function from admission to hospital until the day of ASCT was taken into account. Biological monitoring of renal function took into account blood urea and creatinine. The glomerular filtration rate (GFR) was estimated using the Schwartz formula [23]. Monitoring of liver function took into account transaminases (SGOT, SGPT), gamma-GT, bilirubin, and serum protein. Any neurological symptom occurring during the administration of thiotepa or within seven days (5 half-lives) following the end of the last administration of thiotepa was defined as a NAE. Further examinations distinguished symptoms related to progressive disease or to other causes from NAE. NAE were classified according to the National Cancer Institute Common Terminology Criteria for Adverse Events (version 4.03). Each event detected led to a case analysis to assess the neurological outcome.

Causality assessment of adverse drug reactions (imputability) was performed using the algorithm devised by Begaud et al. which is based on a three-stage process: assessment of chronological criteria, clinical and biological findings and symptom evaluation [24]. The method separates intrinsic imputability (possible cause between drug and clinical event) from extrinsic imputability (bibliographical data) using seven criteria divided in two groups: chronology and semiology (symptoms or signs).

Potential contributing factors were pre-specified and were: (1) sex; (2) age; (3) race; (4) primary tumour site; (5) history of neurological disorders; (6) metabolic disruption; and (7) use of concomitant therapy (antiemetic, antipsychotic, analgesic, antifungals, Proton Pump Inhibitor, and histamine H2-receptor antagonist). Concomitant drugs were determined according to their potential ability to interact with thiotepa metabolism. They were captured the week before, during, and the week after thiotepa exposure.

### Statistical analysis

Some patients had received more than one course of high-dose thiotepa. We decided to retain only one course per patient in order to avoid skewing the statistical analysis by including patients with individual susceptibility. In principle, the first course of high-dose chemotherapy was taken into account in the analysis. The second courses were analyzed separately to study the effect of rechallenge with thiotepa when NAE occurred during the first course. The results are expressed as percentages (qualitative data), or medians and the interquartile range (quantitative data). Concerning the analysis of factors associated with the occurrence of NAE, univariate and multivariable analyses were performed using logistic regression. Variables with a *P* value below 0.05 in the univariate analysis were selected for the multivariable analysis. The results are expressed as the Odds Ratio (OR) of NAE for each factor; an OR equal to 1 is associated with the reference category of each factor. All statistical analyses were two-sided, with *P* values of 0.05 or less deemed statistically significant. The software used was SAS, version 9.1.

## Results

Between May 1987 and March 2011, 307 courses of high-dose thiotepa with ASCT were administered to 251 patients (56 patients received 2 courses of high-dose thiotepa). Baseline characteristics of these 251 children are shown in Table 1. The median age of children was 8 years (IQR 5–15) with a sex ratio of 1.4 boys per 1 girl, and the population was predominantly Caucasian (74%). The total dose of thiotepa per course was 900 mg/m^2^ for 46 patients (18%), 720 mg/m^2^ for 76 patients (30%), and 600 mg/m^2^ for 129 patients (51%). One hundred and sixteen patients (46%) had a primary brain tumour. Twenty children (8%) had a neurological disorder due to disease when they were admitted to hospital: moderate cerebellar syndrome (*n* = 9), central neuropathy (*n* = 1), peripheral neuropathy (*n* = 3), hemiparesis (*n* = 3), and facial paralysis (*n* = 4). Thirty children exhibited early-stage renal failure upon admission to hospital with a median GFR of 80.5 mL/min (IQR, 71–85 mL/min) and a median blood urea level of 4.65 mmol/L (IQR, 3–6.3 mmol/L). The 221 patients without renal insufficiency upon admission to hospital had a median GFR of 123.5 mL/min (IQR, 111–146 mL/min) and a median blood urea level of 3.9 mmol/L (IQR, 2.4–4.6 mL/min). On the day of ASCT, early-stage renal failure had persisted with a median GFR of 79 mL/min (IQR, 68–82 mL/min) and a median blood urea level of 2.6 mmol/L (IQR, 1.7–4.5 mL/min) in 12 of these 30 children. No patients had liver disruption at upon admission to hospital (SGPT median = 26 IU/L, IQR 18–38; SGOT median = 28 IU/L, IQR 22–36; total bilirubin median = 8 μmol/L, IQR 6–10; GGT median = 16 IU/L, IQR 12–24). Thirteen patients had reversible hepatic cytolysis at the end of the thiotepa regimen without cholestasis (SGPT median = 217 IU/L, IQR 177–253; SGOT median = 156 IU/L, IQR 143–179; total bilirubin median = 12 μmol/L, IQR 10–14; GGT median = 17 IU/L, IQR 8–25; serum protein median = 59 g/L, IQR 53–74). During treatment with thiotepa, 92 patients had required analgesics (42%). According to the World Health Organization analgesic ladder, 76 patients (35%) had received simple analgesics such as acetaminophen or nefopam for mild pain, 53 patients (24%) had received weak opioids such as tramadol, codeine, or dextropropoxyphen for mild to moderate pain, and 9 patients (4%) had received strong opioids such as morphine or pethidine for moderate to severe pain. Sometimes, patients had received a combination of different analgesics. Regarding anti-emetic support, every child had received at least a 5-HT3 antagonist. The anti-emetic treatment added was alizapride (43%), or aprepitant (3%). None of the patients had declared an allergy to analgesics or anti-emetic drugs upon admission to hospital.

**Table 1:** Patient characteristics

	Patients (*N* = 251)
Gender	
Boys	145 (58%)
Girls	106 (43%)
Age (years; median [IQR])	8 [5–15]
Race	
Caucasian	186 (74%)
African	40 (16%)
Others	25 (10%)
Brain tumour	116 (46%)
Medulloblastoma	71 (28%)
PNET^a^	26 (10%)
Pinealoblastoma	13 (5%)
ATRT^b^	5 (2%)
Malignant GCT^c^	1 (0**.**4%)
Other tumour site	135 (54%)
Osteosarcoma	50 (20%)
Neuroblastoma	26 (10%)
Ewing tumor	23 (9%)
Rhabdomyosarcoma	20 (8%)
Other sarcoma	4 (2%)
Nephroblastoma	4 (2%)
Burkitt lymphoma	4 (2%)
Hepatoblastoma	2 (1%)
Desmoplastic tumour	2 (1%)
Neurological disorder at baseline^d^	20 (8%)
History of seizure	13 (5%)
Thiotepa dose	
600 mg/m^2^	129 (51%)
720 mg/m^2^	76 (30%)
900 mg/m^2^	46 (18%)
Concomitant drugs^e^	
Alizapride	94 (43%)
Antibiotic	49 (22%)
Antidepressant	7 (3%)
Antipsychotic	40 (18%)
Aprepitant	7 (3%)
Benzodiazepine	43 (20%)
Histamine H2-receptor antagonist	5 (2%)
Proton Pump Inhibitor	7 (3%)
Analgesics	92 (42%)
Simple analgesics	76 (35%)
i.e. acetaminophen, nefopam	
Weak opioids	53 (24%)
i.e. tramadol, codeine, dextropropoxyphen	
Strong opioids	9 (4%)
i.e. morphine, pethidine	

Data are numbers (%), unless otherwise stated

^a^Primitive NeuroEctodermal Tumour

^b^Atypical Teratoid Rhabdoid Tumour

^c^Malignant Germ Cell Tumour

^d^Data missing for one patient

^e^Data missing for 31 patients

**Fig. 1:**
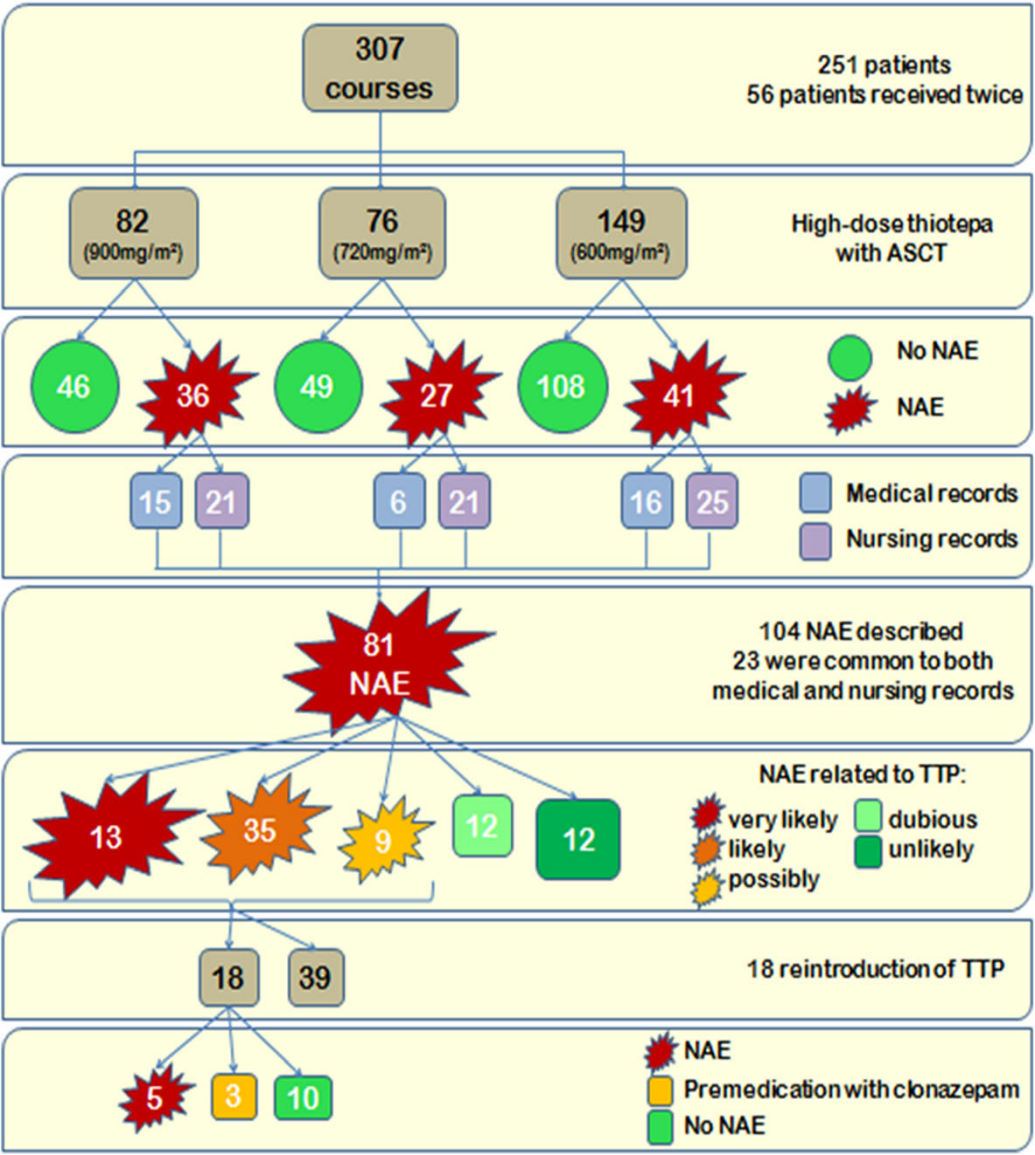
Summary chart of neurological adverse events (NAE)

After reviewing medical and nursing records, 81 NAE (26.4%) were unveiled during the 307 courses, 69 NAE had occurred during the 251 first courses (27.5%), and 12 NAE during the 56 s courses (21.4%). After assessment of intrinsic and extrinsic imputability, NAE were considered “very likely” related to thiotepa for 13 patients (16.0%), “likely” related to thiotepa for 35 patients (43.2%) and “possibly” related to thiotepa for 9 patients (11.1%). Twenty-four NAE (29.6%) were assessed as “dubious” or “unlikely” to be related to thiotepa, and were therefore excluded (Fig. 1). Thus, among the 307 courses of high-dose thiotepa, 57 NAE had occurred (18.6%). Forty-six NAE had occurred during the 251 first courses (18.3%), and 11 during the 56 s courses (19.6%). In this study, the incidence of high-dose thiotepa-related neurotoxicity was therefore estimated at 18.3%. Five children had experienced NAE during the two courses of high-dose thiotepa. Twenty-eight NAE (24%) had occurred in patients with a brain tumour and 18 (13%) in patients with another primary tumour site. None of the 13 patients with hepatic cytolysis had developed NAE.

The median time to the onset of neurological symptoms was 2 days (range 0–4) after the beginning of the course of thiotepa. Table 2 shows the symptoms described for the 46 NAE identified during the first courses of high-dose thiotepa. Some patients had experienced several symptoms. Most of the NAE symptoms were graded 1 or 2, corresponding to a mild or moderate disorder. Headache, dizziness, and confusion, graded 1 or 2, which had occurred in 45 cases (94%), are the neurological symptoms described in the Summary of Product Characteristics of thiotepa. Six events were graded 3: three cases of headache, dizziness, confusion; one case of tremor; one of seizure; and one of cerebellar syndrome. One case of seizure was graded 4 and resulted in life-threatening consequences. All of these events had disappeared without sequelae in a median interval of 3 days (range 1–8 days). Eighteen patients had been rechallenged with thiotepa, and NAE had reappeared in 5 cases. In 3 patients who had experienced seizure during the first course of thiotepa, prophylactic treatment with clonazepam had been administered and no further NAE had occurred. Fig. 1 presents a summary of all NAE-related data observed.

**Table 2:** Description of neurological adverse events (NAE)

	Patients (*N* = 251)					
Symptoms	*n*	NCI CTCAE grade				
		1	2	3	4	5
Headache, dizziness, confusion	48 (15.6%)	23 (47.9%)	22 (45.8%)	3 (6.3%)		
Blurred vision	3 (1.0%)	3 (100%)				
Tremor	8 (2.6%)	3 (37.5%)	4 (50%)	1 (12.5%)		
Neuralgia	2 (0.7%)	1 (50%)	1 (50%)			
Seizure	6 (2.0%)	2 (33.3%)	2 (33.3%)	1 (16.7%)	1 (16.7%)	
Pyramidal tract syndrome	1 (0.3%)		1 (100%)			
Cerebellar syndrome	3 (1.0%)		2 (66.7%)	1 (33.3%)		
Opsoclonus-myoclonus syndrome	1 (0.3%)		1 (100%)			
Coma	1 (0.3%)		1 (100%)			

Table 3 shows details of the univariate analysis performed on the 46 NAE which had occurred during the first courses. A brain tumour (OR, 2.1; 95% CI, 1.1 to 4.0; *P* = 0.03), a neurological disorder at baseline (OR, 3.4; 95% CI, 1.3 to 8.8; *P* = 0.02), alizapride (OR, 2.0; 95% CI, 1.0 to 4·0; *P* = 0.04), and weak opioids (OR, 6.1; 95% CI, 3.0 to 12.4; *P* < 0.0001) were identified as risk factors for thiotepa-related NAE. The weak opioids used were tramadol (*n* = 46), codeine (*n* = 5), and dextropropoxyphen (*n* = 2). NAE had occurred in 21 cases when tramadol had been used (46%; *P* < 0.0001), in two cases with codeine (40%; *P* = 0.26), and in one case with dextropropoxyphen (50%; *P* = 0.29). The risk of NAE was not significantly associated with gender (*P* = 0.41), age (*P* = 0.48), race (*P* = 0.51), a history of seizure (*P* = 0.27), renal failure (*P* = 0.98), or other concomitant drugs. Although not significantly associated with the dose of thiotepa (*P* = 0.18), we observed a trend towards an increasing dose-dependent rate of neurotoxicity (600 mg/m^2^: 14%; 720 mg/m^2^: 22%; 900 mg/m^2^: 24%).

**Table 3:** Univariate and multivariate logistic regression analysis of factors contributing to NAE

	n	NAE (%)	univariate *P*^a^	univariate OR (95% CI)	multivariate *P*^b^	multivariate OR (95% CI)
Gender						
Boys	145	24 (17%)				
Girls	106	22 (21%)	0.41	1.3 (0.7–2.5)		
Age						
No NAE	205	9.3 (6.3)^f^				
Yes NAE	46	8.6 (5.2)^f^	0.48^c^			
Race						
Caucasian	186	31 (17%)				
African	40	9 (23%)	0.51^e^			
Others	25	6 (24%)				
Brain tumour						
No	135	18 (13%)		1		1
Yes	116	28 (24%)	0.03	2.1 (1.1–4.0)	0.04	2.2 (1.0–4.6)
Neurological disorder at baseline						
No	230	38 (17%)		1		1
Yes	20	8 (40%)	0.02	3.4 (1.3–8.8)	0.08	2.7 (0.9–8.4)
History of seizure						
No	238	42 (18%)		1		
Yes	13	4 (31%)	0.27	2.1 (0.6–7.1)		
Thiotepa regimen						
600 mg/m^2^	129	18 (14%)				
720 mg/m^2^	76	17 (22%)	0.18^e^			
900 mg/m^2^	46	11 (24%)				
Alizapride^d^						
No	126	19 (15%)		1		1
Yes	94	25 (27%)	0.04	2.0 (1.0–4.0)	0.13	1.7 (0.8–3.6)
Aprepitant^d^						
No	213	41 (19%)		1		
Yes	7	3 (43%)	0.14	3.1 (0.7–14.6)		
Antipsychotic^d^						
No	180	34 (19%)		1		
Yes	40	10 (25%)	0.39	1.4 (0.6–3.2)		
Proton Pump Inhibitor^d^						
No	213	43 (20%)		1		
Yes	7	1 (14%)	1	0.7 (0.1–5.6)		
Histamine H2-receptor antagonist^d^						
No	215	42 (20%)		1		
Yes	5	2 (40%)	0.26	2.7 (0.4–17)		
Simple analgesics^d^						
i.e. acetaminophen, nefopam						
No	144	20 (13%)		1		
Yes	76	14 (18%)	0.40	1.4 (0.7–3.0)		
Weak opioids^d^						
i.e. tramadol, codeine, dextropropoxyphen						
No	167	20 (12%)		1		1
Yes	53	24 (45%)	0.0001	6.1 (3.0–12.4)	0.0001	6.3 (3.0–13.4)
Strong opioids^d^						
i.e. morphine, pethidine						
No	211	44 (21%)		1		
Yes	9	4 (44%)	0.09	3.0 (0.8–11.8)		

^a^Fisher exact

^b^Taking into account 4 factors: brain tumour (yes/no); neurological disorder at baseline (yes/no); alizapride intake (yes/no); weak opioids (yes/no)

^c^Student *t* test

^d^Data missing for 31 patients

^e^Chi-square test (2 df)

^f^Mean (SD)

In the multivariable analysis, a brain tumour and a weak opioid (tramadol) were identified as independent risk factors for thiotepa-related NAE (Table 3). Patients with a brain tumour were found to have a greater risk of developing NAE than patients with another primary tumour (OR, 2.2; 95% CI, 1.0 to 4.6; *P* = 0.04). The use of tramadol for analgesia was confirmed as a highly significant contributing factor for NAE (OR, 6.3; 95% CI, 3.0 to 13.4; *P* < 0.0001).

## Discussion

In this imputability analysis, the incidence of neurotoxicity due to high-dose thiotepa was estimated at 18.3%. To our knowledge, this is the first study to describe the incidence of neurotoxicity related to high-dose thiotepa, which had been administered below the defined limiting dose (1005 to 1125 mg/m^2^) in all the courses, in accordance with the consensus guidelines [21]. Neurological complications have previously been reported after thiotepa combined with total body irradiation [25–27] or with another cytotoxic agent such as busulfan [28], busulfan and melphalan [29], carboplatin and cyclophosphamide [30], etoposide [31]. It is noteworthy that most children with HDCT receive many other medicines that can cause various neurological signs: opioids and drowsiness, anti-emetics and extrapyramidal syndrome, benzodiazepine and behavioural disorders [32]. Causality studies had then failed to demonstrate unambiguous involvement of thiotepa in the development of neurological disorders. NAE observed in our study are mostly of central nervous system origin with 9.6% of symptoms described as severe (grade 3 or 4). All NAE were reversible and devoid of sequelae, and had not contraindicated rechallenge with high-dose thiotepa.

Various pathomechanisms can explain drug-induced neurological disorders. In this study, we showed that a brain tumour could be an independent risk factor, which could increase the risk for thiotepa-related neurotoxicity 2.2-fold. Drugs with the highest neurotoxicity are therefore those that readily cross the blood-brain barrier. Lipid-soluble agents with a low molecular weight, such as thiotepa and tepa enter the brain easily [32]. Damage to the blood-brain barrier could facilitate the passage of drugs into the brain. Diseases such as malignant brain tumours, which damage the blood-brain barrier, would facilitate the direct neurotoxic effect of such drugs [33, 34].

In our study, we found no influence of renal failure on the occurrence of high-dose thiotepa- related NAE. However, these results should be considered with caution. Only 12 children had renal disruption, classified as an early-stage disorder. Among them, two children had experienced a NAE. Even moderate renal disruption has been described as possibly capable of increasing exposure to thiotepa by 43% and exposure to tepa by 157% [35]. Therefore thiotepa dose adjustments regarding renal clearance remain recommended.

In this study, we showed that intake of a weak opioid, especially tramadol, could be an independent risk factor for thiotepa-related NAE. Drug-related iatrogenia is a major source of treatment failures, especially when multiple medications are used. CYP3A4 and CYP2B6 are the main cytochrome enzymes involved in the metabolism of thiotepa to tepa [12, 13]. Thiotepa was described to be a highly potent, irreversible inhibitor of CYP2B6, which can lead to a risk of a self-induced overdose via a metabolic predisposition of each patient. Caution should be exercised when using thiotepa combined with CYP2B6 substrates, such as tramadol [36, 37]. In addition, aprepitant was described as an CYP3A4 inhibitor which can lead to an overdose of thiotepa [38, 39]. In this study, comedication with aprepitant was not significantly associated with NAE, possibly because of a lack of statistical power (only 3 to 7 patients had been treated with that drug).

Otherwise, interindividual variability in alkylating agent pharmacokinetics may lead to unpredictable toxicity and efficacy. A poor metabolizer phenotype was described as responsible for an increase in exposure to thiotepa and for an increase in the severity of mucositis [40]. This variability is particularly important in cancer therapy due to the narrow therapeutic window of anticancer agents. Interindividual variability in thiotepa clearance has been described as ranging from 28 to 90%, with variability of exposure to tepa after the administration of high-dose thiotepa ranging from 15 to 50% [41]. A pharmacogenetic analysis could therefore be considered before starting high-dose chemotherapy to better predict the metabolizer phenotype [17].

In our study, we have shown that tramadol used concomitantly with high-dose thiotepa could increase the risk of NAE 6.3-fold. Tramadol is a synthetic opiate that is chemically similar to codeine. Its antinociceptive effects are mediated via a combination of the agonist effects of μ-opioid receptors and inhibition of the reuptake of serotonin and norepinephrine [42]. This second property is an important element of analgesia, but also a major element in the risk of developing NAE. Indeed, tramadol is known to lower the seizure threshold and has been reported to be responsible for seizures at therapeutic and toxic doses [43–45]. Similarly, the risk of serotonin syndrome is possible at the usual doses, and seems more common when overused or combined with potent inhibitors of metabolic pathways [46]. The main metabolite of tramadol, which has a 200-fold higher agonist effect for μ-opioid receptors than tramadol, results from the metabolism of tramadol by CYP2D6. However, another metabolic pathway of tramadol, as described above, passes through CYP2B6 and CYP3A4 [47]. Thus, concomitant use of tramadol and high-dose thiotepa, a CYP2B6 inhibitor, could be responsible for an overdose of tramadol, which could promote the emergence of neurological complications via monoaminergic overactivity.

An alternate hypothesis is that there is an unmeasured confounder that leads to both increased risk of NAE and increased need for pain control, hence the observed association with exposure to the weak opiates.

In clinical research, the gold standard is the prospective randomized controlled trial. Retrospective cohort studies, as performed in our study, are in principle subject to multiple risks of bias [48]. Selection bias of patients was reduced or cancelled by the decision to analyze all children with solid tumours treated with high-dose thiotepa followed by ASCT. This bias could therefore only occur if the active file of patients under treatment in the paediatric oncology unit had already been selected simply through admission to the unit. Concerning NAE, it is likely that all qualifying events have not been recorded across the 25 year period, and in particular for low-grade events that may be missing from the records. Regarding interpretation bias, the reading grid was carried out before and the collection was standardized for all patients. Finally, we sought to prevent confounding bias by conducting an analysis of univariate and multivariable logistic regression.

## Conclusions

This study estimates an incidence of 18.3% of high-dose thiotepa-related NAE. These neurological complications resolved without sequelae and rechallenge with thiotepa was possible. A brain tumour and tramadol treatment were found to be possible independent risk factors for thiotepa-related neurotoxicity. Identifying markers for individual susceptibilities is mandatory in order to develop customised treatment approaches for cancer patients treated with intensive chemotherapy.

## Abbreviations

ASCT: Autologous stem cell transplantation
CI: Confidence interval
CSF: Cerebrospinal fluid
GFR: Glomerular filtration rate
GST: Glutathione S-transferase
HDCT: High-dose chemotherapy
IQR: Interquartile range
MTD: Maximum tolerated dose
NAE: Neurological adverse events
OR: Odds Ratio
